# Supplementary material for: Glutamic Acid at Position 168 Is a Constitutive Activator of Tank Binding Kinase 1 Catalytic Function
Source: Neuromolecular Med. 2025 Nov 5;27(1):73. doi: 10.1007/s12017-025-08894-6 (PMC12589330; doi:10.1007/s12017-025-08894-6)
Supplement: Supplementary file 1 — Supplementary Material 1 [file 12017_2025_8894_MOESM1_ESM.pdf]

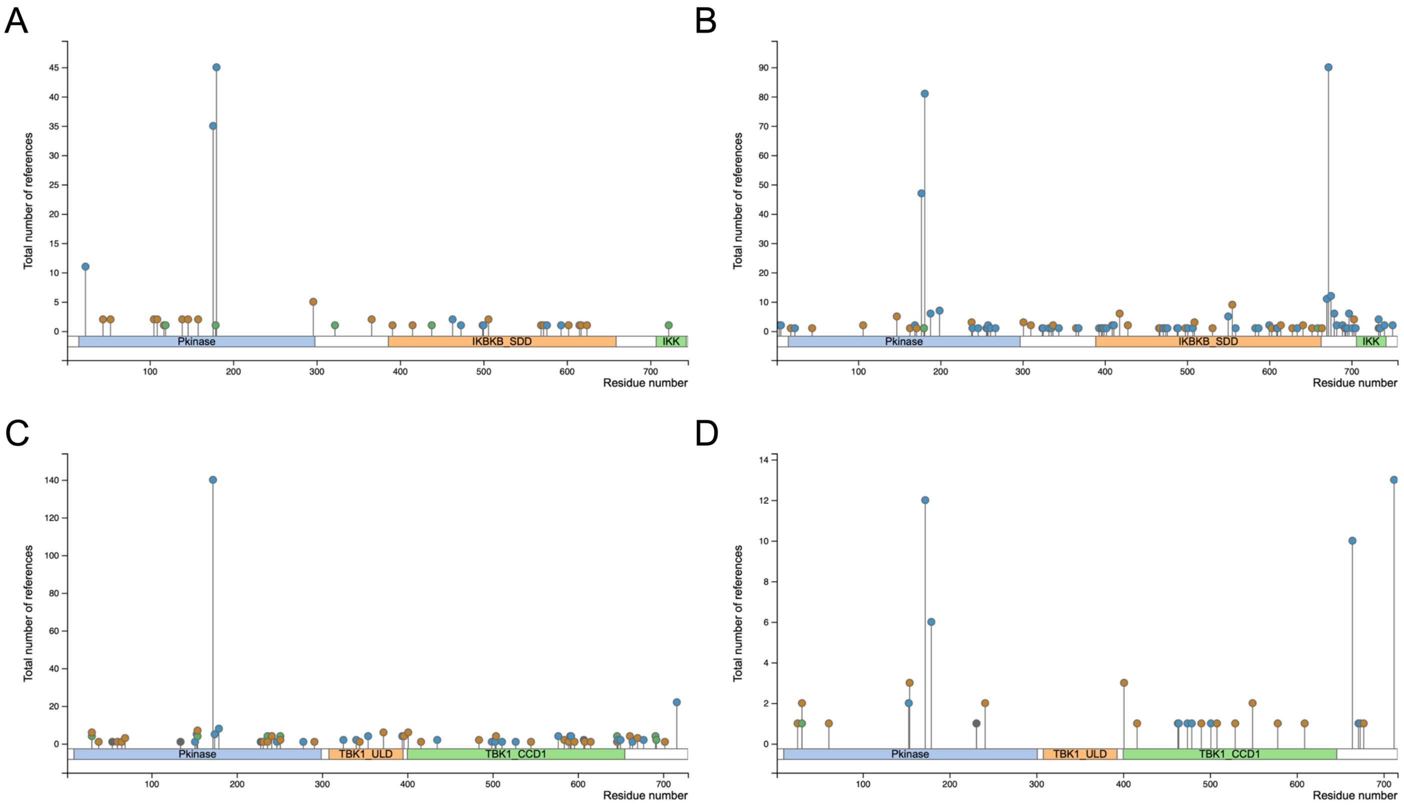

Supplemental figure 1 – Lollipop plot data for posttranslational modifications of IKKA (A), IKKB (B), TBK1 (C), and IKKE (D). The x axis for each plot displays the amino acid open reading frame for each protein from amino to carboxy terminus. Posttranslational modifications are indicated for phosphorylation (●), ubiquitylation (●), and acetylation (●), with the y axis noting the number of references reporting this event.

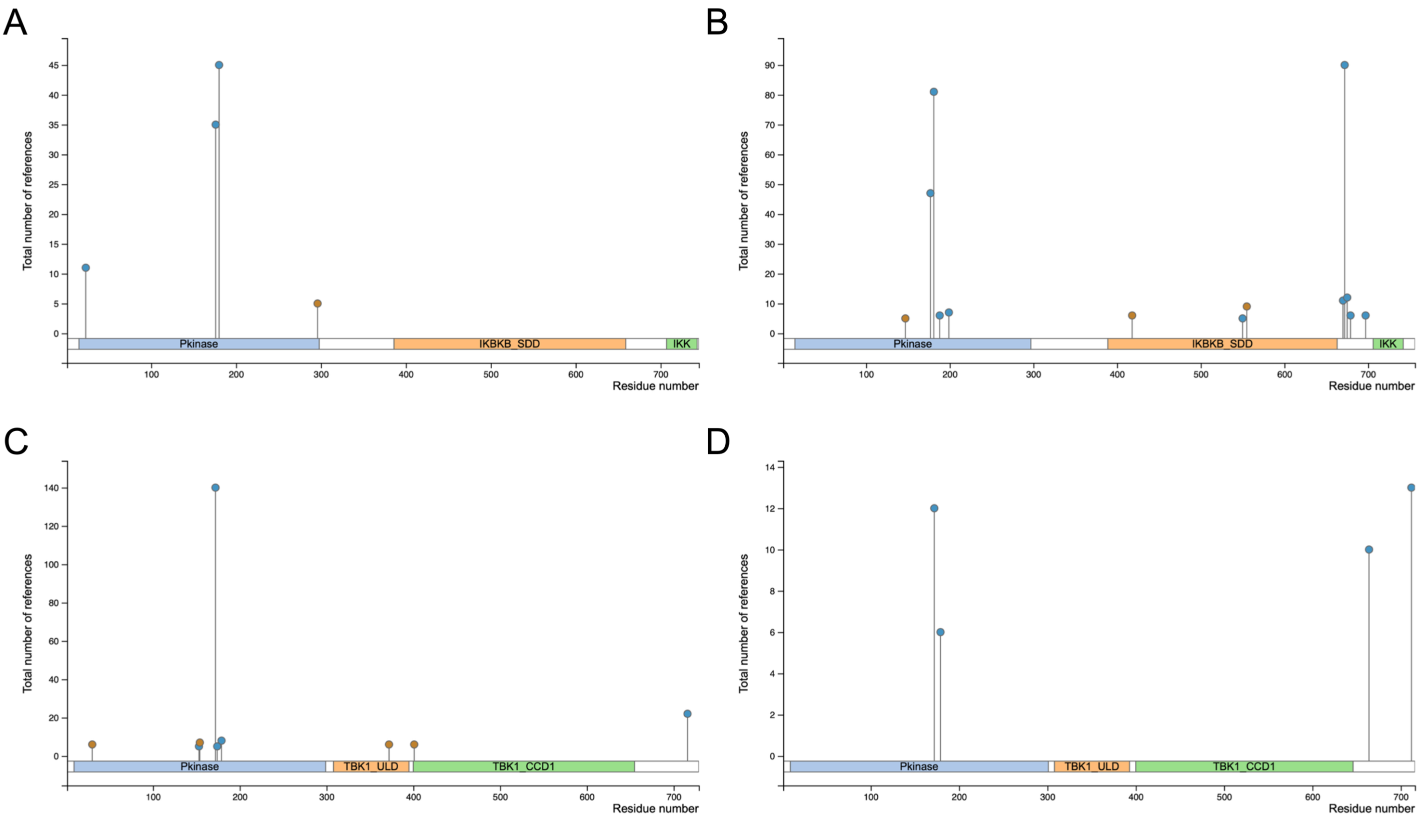

Supplemental figure 2 – Lollipop plot data for posttranslational modifications of IKKA (A), IKKB (B), TBK1 (C), and IKKE (D) with threshold filter applied. The x axis for each plot displays the amino acid open reading frame for each protein from amino to carboxy terminus. Posttranslational modifications are indicated for phosphorylation (●), ubiquitylation (●), and acetylation (●), with the y axis noting the number of references reporting this event. Only posttranslational modifications with ≥5 reports plotted.

Residue	Sequence	HTP	LTP
T23-p	EMRERLGtGGFGNVC	7	4
K44-ub	LDLKIAIkSCRLELS	0	2
K53-ub	CRLELSTkNRERWCH	0	2
K105-ub	CSGGDLRkLLNkPEN	0	2
K109-ub	DLRkLLNkPENCCGL	0	2
K117-ub	PENCCGLkEsQILSL	0	1
S119-ac	NCCGLkEsQILSLLS	1	0
K139-ub	IRYLHENkIIHRDLk	0	2
K146-ub	kIIHRDLkPENIVLQ	0	2
K158-ub	VLQDVGGkIIHKIID	0	2
S176-p	AKDVDQGsLCtsFVG	34	1
T179-ac	VDQGsLCtsFVGTLQ	1	0
S180-p	DQGsLCtsFVGTLQY	45	0
K296-ub	GPVDLTLkQPRCFVL	1	4
K322-ac	ILNMTSAkIISFLLP	0	1
K366-ub	GISLDPRkPASQCVL	0	2
K391-ub	VYLFDKSkTVYEGPF	0	1
K415-ub	NYIVQDSkIQLPIIQ	0	1
K438-ac	VHYVSGLkEDYSRLF	0	1
T463-p	LRYNANLtKMKNTLI	0	2
S473-p	KNTLISAsQQLKAKL	1	0
T499	ERYSEQMTyGISSEk	0	1
Y500-p	RYSEQMTyGISSEkM	0	1
K506-ub	TyGISSEkMLKAWKE	0	2
K569-ub	QRAIDLYkQLkHRPs	0	1
K572-ub	IDLYkQLkHRPsDHS	0	1
S576-p	kQLkHRPsDHSYSDS	0	1
T593-p	MVKIIVHtVQSQDRV	0	1
K602-ub	QSQDRVLkELFGHLS	0	1
K615-ub	LSKLLGCkQkIIDLL	0	1
K617-ub	KLLGCkQkIIDLLPk	0	1
K624-ub	kIIDLLPkVEVALSN	0	1
T722-ac	NCLGHLStIIHEANE	1	0

Supplemental Table 1 – Posttranslational modifications of IKKA, data accessed through the phosphosite portal. Modifications indicated by shading have ≥5 references supporting this event. HTP = high throughput data. LTP = low throughput data.

Residue	Sequence	HTP	LTP
S4-p	____MSWsPsLTTQT	0	2
S6-p	__MSWsPsLTTQTCG	0	2
K18-ub	TCGAWEMkERLGtGG	0	1
T23-p	EMkERLGtGGFGNVI	0	1
K44-ub	TGEQIAIkQCRQELS	0	1
K106-ub	CQGGDLRkYLNQFEN	0	2
K147-ub	RIIHRDLkPENIVLQ	2	3
K163-ub	GEQRLIHkIIDLGyA	1	0
Y169-p	HkIIDLGyAkELDQG	1	1
K171-ub	IIDLGyAkELDQGsL	1	0
S177-p	AkELDQGsLCtsFVG	46	1
T180-p	LDQGsLCtsFVGTLQ	0	1
T180-ac	LDQGsLCtsFVGTLQ	1	0
S181-p	DQGsLCtsFVGTLQy	79	2
Y188-p	sFVGTLQyLAPELLE	6	0
Y199-p	ELLEQQKyTVTVDYW	6	1
K238-ub	WHSKVRQksEVDIVV	1	2
S239-p	HSKVRQksEVDIVVs	0	1
S246-p	sEVDIVVsEDLNGTV	0	1
S256-p	LNGTVKFsssLPyPN	0	1
S257-p	NGTVKFsssLPyPNN	0	1
S258-p	GTVKFsssLPyPNNL	1	1
Y261-p	KFsssLPyPNNLNsV	0	1
S267-p	PyPNNLNsVLAERLE	0	1
K301-ub	YGPNGCFkALDDILN	1	2
K310-ub	LDDILNLkLVHILNM	1	1
T324-p	MVTGTIHtyPVTEDE	0	1
Y325-p	VTGTIHtyPVTEDEs	0	1
S332-p	yPVTEDEsLQsLkAR	0	1
S335-p	TEDEsLQsLkARIQQ	0	1
K337-ub	DEsLQsLkARIQQDt	0	2
T344-p	kARIQQDtGIPEEDQ	0	1
K365-ub	GLALIPDkPAtQCIS	0	1
T368-p	LIPDkPAtQCISDGK	0	1
S393-p	LVFLFDNsKItyEtQ	0	1
T396-p	LFDNsKItyEtQIsP	0	1
Y397-p	FDNsKItyEtQIsPR	0	1
T399-p	NsKItyEtQIsPRPQ	0	1
S402-p	ItyEtQIsPRPQPEs	0	1
S409-p	sPRPQPEsVsCILQE	0	2
S411-p	RPQPEsVsCILQEPk	0	2
K418-ub	sCILQEPkRNLAFFQ	2	4
K428-ub	LAFFQLRkVWGQVWH	1	1
S466	LRNNSCLSkMKNsMA	0	1
K467-ub	RNNSCLSkMKNsMAs	0	1
S471-p	CLSkMKNsMAsMsQQ	0	1
S474-p	kMKNsMAsMsQQLKA	0	1
S476-p	KNsMAsMsQQLKAKL	0	1
T488-p	AKLDFFKtsIQIDLE	0	1
S489-p	KLDFFKtsIQIDLEK	0	1
S498	QIDLEKYSEQTEFGI	0	1
T501	LEKYSEQTEFGITsD	0	1
S507-p	QTEFGITsDkLLLAW	0	1
K509-ub	EFGITsDkLLLAWRE	1	2
K531-ub	CGRENEVkLLVERMM	0	1
S550-p	DIVDLQRsPMGRkQG	0	5
K555-ub	QRsPMGRkQGGtLDD	2	7
T559-p	MGRkQGGtLDDLEEQ	0	1
T583-p	EKPRDQRtEGDsQEM	0	1
S587-p	DQRtEGDsQEMVRLL	0	1
S600-p	LLLQAIQsFEkKVRV	0	2
K603-ub	QAIQsFEkKVRVIyt	0	1
Y609-p	EkKVRVIytQLSkTV	0	1
T610-p	kKVRVIytQLSkTVV	0	1
K614-ub	VIytQLSkTVVCKQK	1	1
K628-ub	KALELLPkVEEVVsL	0	1
S634-p	PkVEEVVsLMNEDEk	0	1
K641-ub	sLMNEDEkTVVRLQE	1	1
K652-ub	RLQEKRQkELWNLLk	0	1
K659-ac	kELWNLLkIACSkVR	0	1
K664-ub	LLkIACSkVRGPVsG	0	1
S670-p	SkVRGPVsGsPDsMN	0	11
S672-p	VRGPVsGsPDsMNAs	0	90
S675-p	PVsGsPDsMNAsRLs	0	12
S679-p	sPDsMNAsRLsQPGQ	1	5
S682-p	sMNAsRLsQPGQLMS	1	1
S689	sQPGQLMSQPStAsN	1	1
S692	GQLMSQPStAsNsLP	1	0
T693-p	QLMSQPStAsNsLPE	0	1
S695-p	MSQPStAsNsLPEPA	0	1
S697-p	QPStAsNsLPEPAkK	1	5
P701	AsNsLPEPAkKSEEL	1	0
K703-ub	NsLPEPAkKSEELVA	2	2
S705	LPEPAkKSEELVAEA	1	0
S733-p	TVREQDQsFtALDWs	2	2
S733-gl	TVREQDQsFtALDWs	1	0
T735-p	REQDQsFtALDWsWL	0	1
S740-p	sFtALDWsWLQTEEE	1	1
S750-p	QTEEEEHsCLEQAS_	2	0

Supplemental Table 2 - Posttranslational modifications of IKKB, data accessed through the phosphosite portal. Modifications indicated by shading have ≥5 references supporting this event. HTP = high throughput data. LTP = low throughput data.

Residue	Sequence	HTP	LTP
K30-ac	VFRGRHKkTGDLFAI	2	2
K30-ub	VFRGRHKkTGDLFAI	4	2
K38	TGDLFAIKVFNNISF	0	1
R54-me	RPVDVQMrEFEVLkK	1	0
K60-ub	MrEFEVLkKLNHkNI	0	1
K65-ub	VLkKLNHkNIVkLFA	0	1
K69-ub	LNHkNIVkLFAIEEE	1	2
R134-me	RENGIVHrDIKPGNI	1	0
S151-p	VIGEDGQsVykLTDF	0	1
Y153-p	GEDGQsVykLTDFGA	1	4
K154-ac	EDGQsVykLTDFGAA	2	2
K154-ub	EDGQsVykLTDFGAA	1	6
S172-p	EDDEQFVsLyGTEEy	87	53
Y174-p	DEQFVsLyGTEEyLH	1	4
Y179-p	sLyGTEEyLHPDMYE	3	5
R228-me	FRPFEGPrRNkEVMY	1	0
K231-ub	FEGPrRNkEVMYkII	0	1
K236-ac	RNkEVMYkIITGkPS	2	2
K236-ub	RNkEVMYkIITGkPS	0	1
K241-ac	MYkIITGkPSGAIsG	2	2
K241-ub	MYkIITGkPSGAIsG	0	4
S247-p	GkPSGAIsGVQkAEN	0	1
K251-ac	GAIsGVQkAENGPID	2	2
K251-ub	GAIsGVQkAENGPID	0	2
T278-p	RGLQVLLtPVLANIL	0	1
K291-ub	ILEADQEkCWGFDQF	0	1
Y325-p	QMTAHKIyIHSYNTA	1	1
Y340-p	TIFHELVyKQTkIIS	1	1
K344-ub	ELVyKQTkIISSNQE	0	1
Y354-p	SSNQELIyEGRRLVL	1	3
K372-ub	RLAQHFPkTTEENPI	3	3
Y394-p	LNTIGLIyEkISLPk	2	2
K396-ub	TIGLIyEkISLPkVH	0	4
K401-ub	yEkISLPkVHPRYDL	4	2
K416-ub	DGDASMAkAITGVVC	0	1
Y435-p	IASTLLLyQELMRKG	1	1
K484-ub	KTVKVYEkLMKINLE	0	2
S499	AAELGEISDIHtkLL	0	1
T503-p	GEISDIHtkLLRLSS	0	1
K504-ub	EISDIHtkLLRLSSs	0	4
S511-p	kLLRLSSsQGTIETS	0	1
S527-p	QDIDSRLsPGGSLAD	1	0
K545-ub	HQEGTHPkDRNVEKL	0	1
Y577-p	KAERRLAyNEEQIHk	2	2
K584-ub	yNEEQIHkFDKQkLy	0	2
K589-ub	IHkFDKQkLyyHATk	0	1
Y591-p	kFDKQkLyyHATkAM	2	2
Y592-p	FDKQkLyyHATkAMT	2	2
K596-ub	kLyyHATkAMTHFTD	0	1
K607-ac	HFTDECVkkYEAFLN	1	1
K607-me	HFTDECVkkYEAFLN	1	1
K608-ub	FTDECVkkYEAFLNk	0	1
K615-ub	kYEAFLNkSEEWIRK	0	1
K646-ac	DIEEEVSkyQEyTNE	2	2
K646-ub	DIEEEVSkyQEyTNE	0	1
Y647-p	IEEEVSkyQEyTNEL	1	1
Y650-p	EVSkyQEyTNELQET	1	1
K661-ub	LQETLPQkMFtASSG	1	3
T664-p	TLPQkMFtASSGIkH	0	1
K670-ub	FtASSGIkHTMTPIy	2	1
Y677-p	kHTMTPIyPSSNTLV	1	1
K691-ac	VEMTLGMkkLKEEME	2	2
K692-ac	EMTLGMkkLKEEMEG	1	1
K702-ub	EEMEGVVkELAENNH	0	1
S716-p	HILERFGsLTMDGGL	2	20

Supplemental Table 3 - Posttranslational modifications of TBK1, data accessed through the phosphosite portal. Modifications indicated by shading have ≥5 references supporting this event. HTP = high throughput data. LTP = low throughput data.

Residue	Sequence	HTP	LTP
K25-ub	GATASVYkARNKkSG	0	1
K30	VYkARNKKSGELVAV	0	1
K30-ub	VYkARNKkSGELVAV	1	1
K61-ub	REFEVLRkLNHQNIV	0	1
Y153-p	GEEGQSIykLTDFGA	1	1
K154-ub	EEGQSIykLTDFGAA	0	3
S172-p	DDDEKFVsVYGTEEy	8	4
Y179-p	sVYGTEEyLHPDMYE	1	5
K231-ub	FGGPRRNkEIMYRIT	0	1
K231-sm	FGGPRRNkEIMYRIT	1	0
K241-ub	MYRITTEkPAGAIAG	0	2
K401-ub	DPALDVPkFVPKVDL	1	2
K416-ub	QADYNTAkGVLGAGY	0	1
T463-p	RTLEVARtsLLYLSS	0	1
S464-p	TLEVARtsLLYLSSS	0	1
T474-p	YLSSSLGtERFSsVA	0	1
S479-p	LGtERFSsVAGTPEI	0	1
K490-ub	TPEIQELkAAAELRS	0	1
T501-p	ELRSRLRtLAEVLSR	1	0
R508	tLAEVLSRCSQNITE	0	1
K529	SLNRELVKSRDQVHE	0	1
K549-ub	QIQCCLDkMNFIYKQ	0	2
K578-ub	EQIHKLDkVNFSHLA	0	1
K609-ub	ASLVTHGkRMRVVHE	0	1
S664-p	RAKGAQAsPPPIAPy	0	10
Y671-p	sPPPIAPyPsPTRkD	0	1
S673-p	PPIAPyPsPTRkDLL	0	1
K677-ub	PyPsPTRkDLLLHMQ	0	1
A712	ERLNRVPAPPDV___	0	13

Supplemental Table 4 - Posttranslational modifications of IKKE, data accessed through the phosphosite portal. Modifications indicated by shading have ≥5 references supporting this event. HTP = high throughput data. LTP = low throughput data.
